# Identification of wheat stress-responding genes and *TaPR-1-1* function by screening a cDNA yeast library prepared following abiotic stress

**DOI:** 10.1038/s41598-018-37859-y

**Published:** 2019-01-15

**Authors:** Jingyi Wang, Xinguo Mao, Ruitong Wang, Ang Li, Guangyao Zhao, Jinfeng Zhao, Ruilian Jing

**Affiliations:** National Key Facility for Crop Gene Resources and Genetic Improvement/Institute of Crop Science, Chinese Academy of Agricultural Sciences, Beijing, 100081 China

## Abstract

Abiotic stress significantly impacts growth and yield of crop plants. It is imperative for crop improvement to discover and utilize stress-tolerant functional genes. In this study, genes responding to abiotic stresses, such as freezing, salt and osmotic stress, were screened from a cDNA yeast library that was constructed from the drought- and heat-tolerant wheat variety Hanxuan 10. After screening for surviving clones we isolated 7,249, 4,313 and 4,469 raw sequences, corresponding to 4,695, 2,641 and 2,771 genes following each treatment. Venn diagrams revealed 377 overlapping genes. GO analysis suggested that these genes were mainly involved in the metabolic and stress signal pathways. KEGG pathway enrichment analysis indicated that the isolated genes predominantly belonged to pathways concerning energy and metabolism. Overlapping gene *TaPR-1-1* within the pathogenesis-related (PR) protein family was selected for detailed characterization. Although previous studies had shown that *PR* genes function during pathogen attack, our results demonstrated that *TaPR-1-1* expression was also induced by freezing, salinity, and osmotic stresses. Overexpression in yeast and *Arabidopsis* showed that *TaPR-1-1* conferred tolerance to these stresses. We concluded that screening cDNA yeast libraries following abiotic stress is an efficient way to identify stress-tolerance genes.

## Introduction

Abiotic stresses, such as salinity, drought and freezing, significantly impact wheat production^1,2^. The Food and Agriculture Organization (FAO) estimates are that the demand for food will increase greatly by 2050^3^. Therefore, identification of functional genes that respond to stress has become an important research objective and this also could provide sources of stress resistance for breeding programs^4,5^. During the last few decades numerous functional factors thought to have a role in abiotic stress response have been isolated; these include components of the SOS (Salt Overly Sensitive) pathway, abscisic acid (ABA) pathway, kinases, phosphatases, ion transporters, and transcription factors^6–8^.

Methods for isolating genes include map-based cloning, yeast two hybrid analysis, transcriptomics analysis, proteomics analysis, biochemical methods, and cloning by homology^9–11^. Screening of yeast libraries that express heterologous cDNA is also an effective method to identify functional genes^12–15^. Because cellular responses to stress are conserved in eukaryotes, yeast is a much easier model for genetic research than plants, and heterologous proteins in yeast are biologically active, and undergo similar behavior to plants in respect of protein folding and glycosylation^16,17^. According to Zhu^18^ stress signaling pathways in plants evolved from yeast and mammalian energy sensing indicating that many stress tolerance components should be conserved.

Several genes involved in abiotic stress response were identified by screening plant cDNA libraries expressed in *E*. *coli*, *Agrobacterium* or yeast. For example, a mannose-1-phosphate guanyl transferase gene from a rice cDNA yeast library was identified as source of salinity response, the *Dbf2* kinase gene from an *Arabidopsis* cDNA yeast library was identified as an essential part of stress tolerance, and SR-like splicing proteins were isolated from a cDNA yeast *Arabidopsis* library developed following salt treatment^19–22^. However, there are no similar reports for wheat and wheat cDNA yeast libraries are needed to identify stress-related genes. Similar studies in other plant species have not reported enrichment analyses for genes detected in screens. With release of the wheat genome sequence, screening cDNA yeast libraries to identify wheat stress-tolerance genes is feasible^23–25^.

Pathogenesis-related protein (PR-1) was first discovered in tobacco leaves infected by tobacco mosaic virus^26^. Some studies showed that PR protein participated in disease resistance- responses mediated by salicylic acid^27,28^. Other studies suggested that PR proteins also function in abiotic stress. For example, AtPR protein had a role in seed germination under salt stress^29^, and AtPR1, AtPR2, and AtPR5 functioned in response to drought stress^30^. Rice pathogenesis-related 1a protein/sperm coating protein (OsSCP) enhanced abiotic stress tolerance^31,32^. Spinach and peanut PR10 had a role in the stress signaling pathway^33–35^. These studies indicated that PR proteins function not only in responses to biotic stress, but also in response to abiotic stress. There are 23 cloned *TaPR-1* genes in wheat, and all contain intron-free open reading frames^36,37^. Their functions are not very clear.

In the present research stress-related genes screened from wheat cDNA yeast libraries following three separate abiotic treatments were subjected to GO and KEGG analyses. *TaPR-1-1*, one of the genes responding to all three treatments was selected for further investigation. Overexpression of *TaPR-1-1* in yeast and *Arabidopsis* revealed various functions in stress tolerance.

## Materials and Methods

### Construction of the wheat cDNA yeast library

Drought and heat tolerant common wheat variety Hanxuan 10 was used as the plant material for construction of the cDNA yeast library. Two-week-old Hanxuan 10 seedlings were separately treated at low temperature (4 °C), 250 mM NaCl, 16.1% PEG6000, and sprayed with 50 μM ABA solution. Seedling samples were harvested at 0, 0.5, 1, 3, 6, 12 and 24 h post treatments. Total RNAs were extracted with Trizol reagent (Invitrogen, 15596-018). After pooling equal quantities of RNAs from each treatment the pooled mRNA was purified with a FastTrack MAG mRNA Isolation Kit (Invitrogen, K1580-02). The ‘SuperScript III First-Strand Synthesis System’ (Invitrogen, 18080-051) was used for reverse transcription. A wheat cDNA library was prepared using a CloneMiner II cDNA Library Construction Kit (Invitrogen, A11180) following the manufacturer’s instructions. Three kinds of reading frame adapters (adapter information is provided in Supplementary Table S1) were ligated to the 5′ ends of the cDNA to ensure correct translation. After introduction into a pDONR222 vector by the Gateway BP reaction, the cDNA inserts were recombined into a pGADT7-DEST vector by LR reaction, in which the insertions were driven by the ADH1 promoter. Finally, the library plasmids were isolated, purified and transformed into yeast strain Y187 using the PEG/LiAc method.

### Screening the wheat cDNA yeast library

A pilot experiment of the three treatments was carried out to determinate the screening conditions. For the freezing treatment, the cDNA library yeast and control yeast transformed with empty pGADT7 (AD) vector were diluted with SD/-Leu liquid medium, spread on an SD/-Leu medium plate, and treated at −20 °C for 3, 6, 7 or 8 days, and incubated at 30 °C for 3 days. For salinity treatment, the cDNA library yeast and control yeast were incubated on SD/-Leu medium supplemented with 1.2, 1.4, 1.6 or 1.8 M NaCl for 5 days. For osmotic stress treatment, the cDNA library yeast and control yeast were incubated on SD/-Leu medium supplemented with 3.2, 3.4, 3.5 or 3.6 M sorbitol for 5 days. The final screening conditions were as follows: for the freezing treatment the library was incubated on SD/-Leu medium at 30 °C for 3 days following treatment at −20 °C for 8 days; for salinity treatment, the library was incubated on SD/-Leu medium supplemented with 1.8 M NaCl for 5 days; for osmotic stress treatment, the library was incubated on SD/-Leu medium supplemented with 3.6 M sorbitol for 5 days.

### Isolation of stress-related genes

Clones surviving after treatment were selected for PCR using pGADT7 vector primer T7 and 3′ AD. The PCR products were purified and sequenced by T7 sequencing primer in an ABI3730 DNA analyzer. This provided raw sequence data for the stress-related genes.

### GO and KEGG enrichment analysis

Clean sequences were obtained after removing poor quality sequences, empty vector regions, poly A regions, and incorrectly translated sequences from the data (Qv < 15). The sequences were then mapped to the Release-32 version of the wheat reference genome (ftp://ftp.ensemblgenomes.org/pub/release-32/plants/fasta/triticum_aestivum/) using the blat tool, and corresponding genes were detected in the wheat genome database. Venn diagrams were made to show the numbers of genes isolated following each treatment. The GO terms for isolated genes were extracted (http://www.geneontology.org). KEGG pathways for the isolated genes were retrieved (http://www.kegg.jp/kegg/kegg1.html) and R function ‘phyper’ was used for enrichment analysis^38–41^.

### Phenotypic analysis of *TaPR-1-1*/*TaMYC2*/*TaHSP70* transgenic yeast

*TaPR-1-1 (TRIAE_CS42_5BL_TGACv1_404207_AA1290140), TaMYC2 (TRIAE_CS42_4DL_TGACv1_344028_AA1142670) and TaHSP70 (TRIAE_CS42_5AL_TGACv1_374650_AA1205560)* full length cDNA were separately cloned into the pGBKT7 (BD) vector all at the *Nde* I and *Bam*H I enzyme sites and transformed to yeast strain AH109 (Supplementary Table S2). Transformed yeast was incubated on SD/-Trp medium at 30 °C for 3 days following exposure to −20 °C for 8 days, on SD/-Trp medium supplemented with 1.8 M NaCl for 5 days, or on SD/-Trp media supplemented with 3.6 M sorbitol for 5 days. Yeast harboring an empty BD was used as the control.

### Phylogenetic tree construction of TaPR-1-1

Various pathogenesis-related protein 1 genes were obtained for different plant species by protein BLAST analysis (http://blast.ncbi.nlm.nih.gov/Blast.cgi). A phylogenetic tree was constructed by the maximum likelihood method in Molecular Evolutionary Genetics Analysis (MEGA) software version 5.2.

### Expression analysis of the *TaPR-1-1* gene in wheat seedlings

Stress-treated samples and cDNA used in constructing the wheat cDNA yeast library were used to detect responses of the *TaPR-1-1* gene to abiotic stress. Primers were designed using Primer Premier 5 software (Supplementary Table S3). Real-time PCR was carried out on an ABI QuantStudio® 7 Flex using the SYBR Green PCR master mix kit (TaKaRa, DRR820A) according to the manufacturer’s instructions^42^. The *GAPDH* gene was used as the internal control. Relative expression values were calculated using the 2−△△*C*_T_ method. For each sample, three biological replicates and three technical replicates were tested.

### Transgenic *Arabidopsis* lines containing *TaPR-1-1*

The full length cDNA of *TaPR-1-1* was fused into a vector pCAMBIA1300 modified at the *Spe* I and *Kpn* I enzyme sites with a 35S promoter (Supplementary Table S2). The fusion vector and empty vector were transformed into *Arabidopsis* by the *Agrobacterium*-mediated floral dip method to obtain stable transgenic lines^43^. Hygromycin was used to select transformed lines. Seeds of homozygous T3 lines were used for further study.

### Phenotypic analyses of transgenic *Arabidopsis* lines

Surface-sterilized *Arabidopsis* seeds were placed on MS medium (Sigma-Aldrich, M5519) with 3% (w/v) sucrose and 0.8% agar in plates and cultured in an incubator with 16 h light (21 °C)/8 h darkness (19 °C).

#### Freezing tolerance assays

Freezing tolerance assays were performed as described with some modifications^44^. Two-week old *Arabidopsis* seedlings were treated with cold acclimation at 4 °C for 2 days and were then placed in a freezing chamber at −10 °C for 12 h. After freezing treatment, the seedlings were transferred to 4 °C in the dark for 12 h, and then were incubated with 16 h light (21 °C)/8 h darkness (19 °C). After 3 days of recovery, survival rates were recorded. Ion leakage was determined following boiling. Briefly, 1 g of two-week-old seedling tissue was placed in 10 ml water, and conductivity of the liquid was measured after shaking for 30 min. Conductivity was measured again after the solution was boiled for 10 min. Ion leakage was measured as the ratio of conductivity before and after boiling.

#### Salinity tolerance assays

Seven-day-old *Arabidopsis* seedlings were transferred to MS solid medium containing 200 mM NaCl and grown for an additional 7 days in vertical culture. Main root lengths were recorded and chlorophyll contents were measured as follows: the seedlings were harvested, weighed and ground into powder in liquid nitrogen; chlorophyll was extracted in *N*,*N*-dimethylformamide (DMF) followed by centrifugation, and absorbance of the supernatant was measured using a spectrophotometer with wavelength settings of 646.8 nm and 663.8 nm, and normalized to fresh weight^45^.

#### Osmotic tolerance assays

Surface sterilized seeds were planted on MS medium and MS medium supplemented with 300 mM sorbitol. Green cotyledon ratios were scored after 7 days.

## Results

### Selection of stress conditions for screening cDNA yeast library

The yeast library diluted 100,000 times and incubated on SD/-Leu media yielded 303 clones, i.e. the colony forming unit (CFU) of the cDNA library was 3.0 × 10^7^ cells/ml (Supplementary Fig. S1a). The sizes of cDNA inserts ranged from 500 to 5,000 bp (Supplementary Fig. S1b). The average insert length was 1,517 bp, longer than reported wheat average transcript length of 1,405 bp^46^. The empty vector rate was 4.3%. The library was assessed to be of suitable quality for screening of functional genes.

Abiotic stress-related genes were isolated from the wheat cDNA yeast library, three stress conditions were optimized and imposed, including the freezing, salinity (NaCl) and osmotic (sorbitol) treatments. Yeast survival rates declined with longer periods of freezing, and higher concentrations of NaCl and sorbitol (Supplementary Fig. S2). Using 10% survival rate of library yeast and no survival for the control transformed with empty vector as the optimal, the following screening conditions were chosen. For freezing stress the yeast library was selected on SD/-Leu medium after 8 days at −20 °C. For salinity stress the yeast library was isolated on SD/-Leu medium supplemented with 1.8 M NaCl, and for osmotic stress the yeast library was screened on SD/-Leu medium supplemented with 3.6 M sorbitol.

### Venn diagram summarizing the response genes to abiotic stress

**Figure 1:**
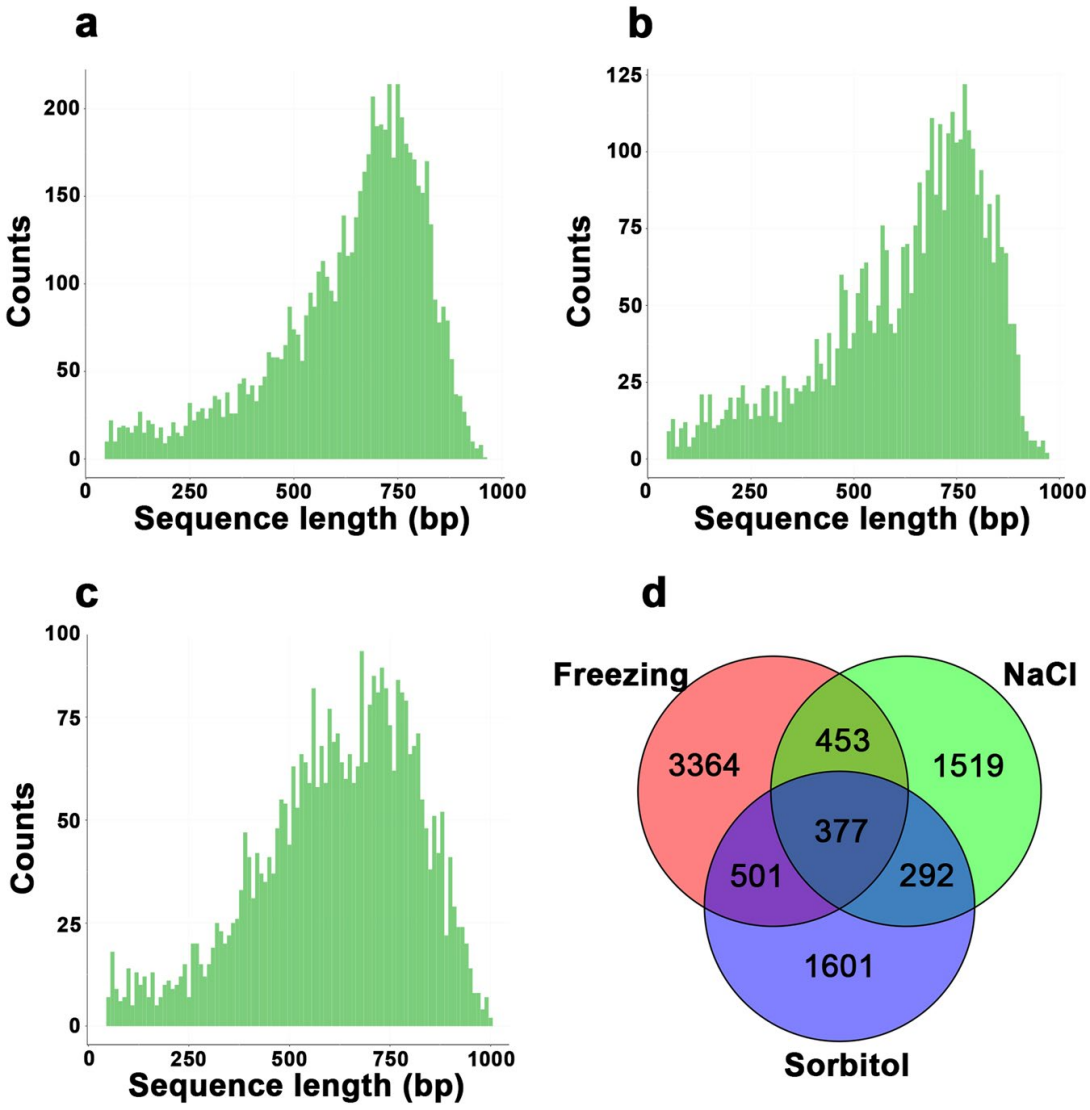
Sequence length distributions and Venn diagram. (**a**–**c**) Isolated sequence length distribution of cDNA fragments from libraries following (**a**) freezing treatment library, (**b**) salinity treatment and (**c**) osmotic stress treatment library. (**d**) Venn diagram showing overlapping genes isolated from the three stress treatments. Numbers in each circle indicate total numbers of isolated genes in the indicated treatment, and the numbers in overlapping areas are the numbers of shared genes.

Around 10 million yeast clones were screened. Most of the clones were dead or showed abnormal growth, whereas less than 10% maintained normal growth. After the freezing treatment, more than 10,000 surviving clones were selected for PCR with T7 and 3′ AD primer. A total of 7,249 raw sequences with size of more than 500 bp were obtained using T7 sequencing primer. Likewise, after salinity and osmotic stress treatments, 4,313 and 4,469 raw sequences, respectively, were obtained. After removing poor quality sequences (Qv < 15), empty vector regions, poly A regions, and incorrectly translated sequences, 6,697, 4,031 and 3,736 clean sequences were obtained from each treatment library. As shown in Fig. 1a–c, sequence lengths ranged from 50 bp to 1,000 bp based on Sanger sequencing. All sequences were less than 1,000 bp and >75% of sequences exceeded 500 bp. Each library consisted of 6,632, 3,976 and 3,697 sequences, which were mapped to the Release-32 version wheat reference genome (ftp://ftp.ensemblgenomes.org/pub/release-32/plants/fasta/triticum_aestivum/). Hence >98.7% of all sequences were mapped to the wheat genome; 6,108, 3,643 and 3,407 sequences had corresponding genes, and 4,695, 2,641 and 2,771 genes in the wheat genome database were detected within each library. Of the matches 2,056 (33.7%), 1,104 (30.3%) and 1,112 (32.6%) sequences contained full length CDS (coding sequences) including start codons, and 3,726 (61.0%), 2,274 (62.4%), and 2,247 (66.0%) sequences contained CDS stop codons. Some CDS stop codons were not sequenced probably due to using vector primer T7 as sequencing primer and max Sanger sequence length is about 1000 bp. The Venn diagram indicated that there were 377 overlapping genes in these three cDNA libraries (Fig. 1d). Under freezing 28.3% of the isolated genes overlapped with those captured under from the other two treatments; under NaCl treatment, 42.5% of isolated genes overlapped with those isolated following the other treatments; and under osmotic stress, 42.2% genes isolated overlapped with those detected following the other two treatments. This meant that many genes responded to multiple stress treatments.

After sequencing of 7,249, 4,313, and 4,469 clones, 4,695, 2,641 and 2,771 genes were isolated from the respective libraries following freezing, salinity and osmotic stress treatments. The relationships between clone number and gene number for the three treatments are shown in Supplementary Fig. S3; and all *R*^2^ were >0.93. On checking the last 100 sequenced clones, 60–70% of sequences were similar to clones already obtained. This indicated that most stress-related genes had been captured from these libraries. Under freezing, NaCl, and sorbitol treatments, the most frequently screened sequence was *TRIAE_CS42_U_TGACv1_642768_AA2122990* detected in 28, 25 and 21 clones, respectively.

### GO terms and KEGG pathway enrichment of screened genes

**Figure 2:**
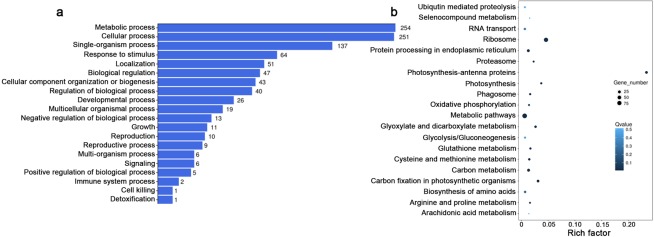
GO and KEGG analysis of genes responding to all three stress treaments. (**a**) Biological processes of isolated genes. (**b**) KEGG pathway enrichment of isolated genes. KEGG pathways for the isolated genes were retrieved (http://www.kegg.jp/kegg/kegg1.html)^59,60^. The rich factor reflects the proportion of isolated genes in a given pathway. Circle areas represent the relative numbers of isolated genes in the pathway; circle colors represent the range of Q values.

In GO annotation, the isolated genes from the freezing-treated library were distributed across 22 biological processes, 20 cellular components, and 12 molecular functions; isolated genes from the salinity treated library were distributed across 23 biological processes, 15 cellular components, and 12 molecular functions; and isolated genes from the osmotic treated library were distributed in 23 biological processes, 16 cellular components, and 12 molecular functions. Overlapping genes from the three libraries were distributed across 20 biological processes, 14 cellular components, and 8 molecular functions (Supplementary Figs S4–S7). The biological processes of genes isolated from the three treatment libraries were predominantly involved in metabolic processes, cellular processes, single-organism processes, and response to stimuli (Fig. 2a). Furthermore, KEGG pathway enrichment analyses showed the top 20 enriched pathways (Supplementary Figs S8–S10). Among 377 overlapping genes, ribosome, photosynthesis, carbon fixation in photosynthetic organisms, glyoxylate and dicarboxylate metabolism, carbon metabolism, metabolic pathways, glycolysis/gluconeogenesis, and oxidative phosphorylation pathway were enriched (Fig. 2b). These pathways mainly related to energy and metabolism already documented to have roles in stress tolerance. Additionally, ubiquitin mediated proteolysis and proteasome pathways were also enriched suggesting that protein degradation was a common response to stress.

### Overexpression of *TaPR-1-1*/*TaMYC2*/*TaHSP70* in yeast confers enhanced multiple abiotic stress tolerance

**Figure 3:**
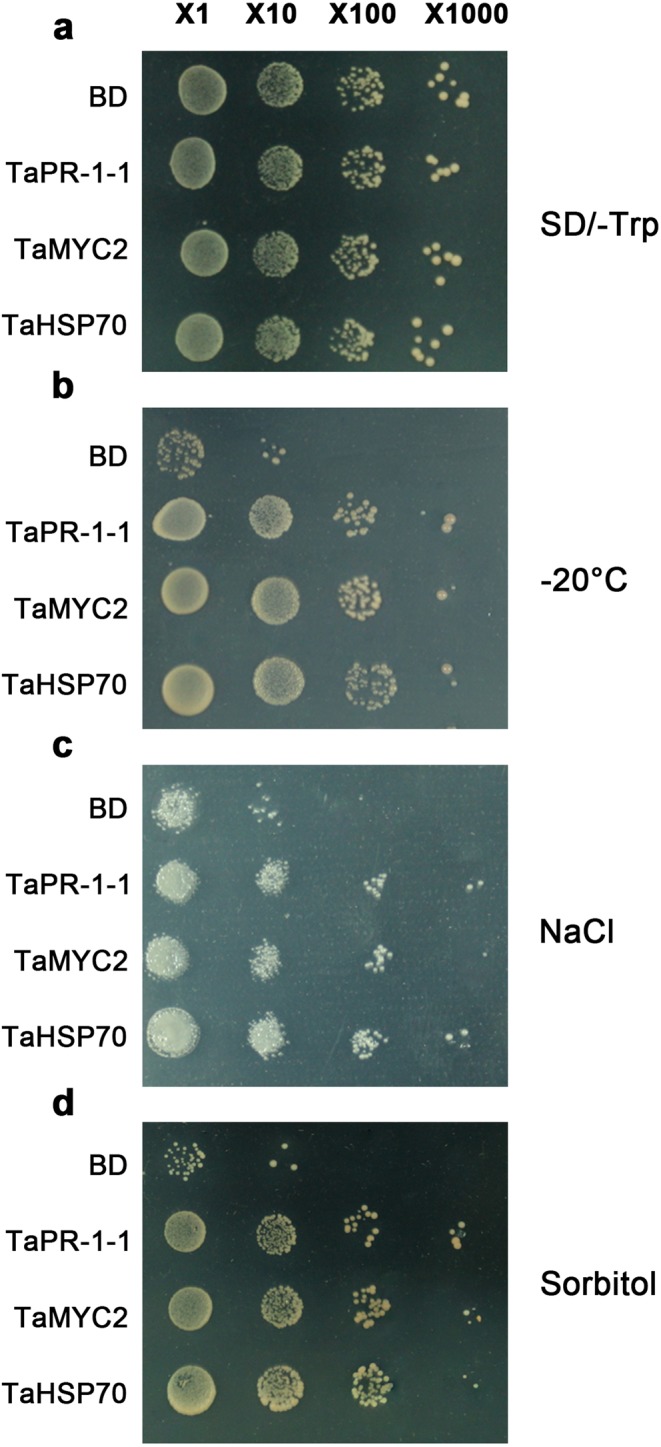
Overexpression of *TaPR-1-1*/*TaMYC2*/*TaHSP70* in yeast enhances multiple abiotic stress tolerance. (**a**) Empty pGBKT7 vector (BD) and *TaPR-1-1*/*TaMYC2*/*TaHSP70* transformed into AH109 grown on SD/-Trp medium for 3 days. (**b**) BD and *TaPR-1-1*/*TaMYC2*/*TaHSP70* transformed into AH109 grown on SD/-Trp media for 3 days after −20 °C treatment for 8 days. (**c**) BD and *TaPR-1-1*/*TaMYC2*/*TaHSP70* transformed into AH109 grown on SD/-Trp medium supplemented with 1.8 M NaCl for 5 days. (**d**) BD and *TaPR-1-1*/*TaMYC2*/*TaHSP70* transformed into AH109 grown on SD/-Trp media supplemented with 3.6 M sorbitol for 5 days. ‘×1’ indicates without dilution, ‘×10’ indicates with 10^−1^ dilution, ‘×100’ indicates with 10^−2^ dilution, ‘×1000’ indicates with 10^−3^ dilution.

Three overlapping genes, *TaPR-1-1*, *TaMYC2* and *TaHSP70*, were selected to validate the accuracy of screening genes from the cDNA yeast library. PR protein had been shown previously to participate in abiotic stress response. MYC2 is a basic helix-loop-helix (bHLH) leucine zipper transcription factor; its expression can be ABA-induced and it takes part in the stress signal response pathway. Abiotic stresses usually cause protein dysfunction, while heat-shock protein (HSP) assists in protein refolding under stress conditions. The three genes were separately cloned into pGBKT7 vector with an ADH1 promoter, and transformed into yeast strain AH109, hence different from the AD vector and Y187 yeast strain used in the library screening. As shown in Fig. 3, there was no obvious difference in growth on SD/-Trp medium between yeasts separately overexpressing the three genes and control yeast transformed by BD empty vector (Fig. 3a). Exposed to −20 °C for 8 days, 1.8 M NaCl for 5 days, or 3.6 M sorbitol for 5 days, the transgenic yeasts showed significant survival, whereas the controls were dead (Fig. 3b–d). The results demonstrated *TaPR-1-1*, *TaMYC2* and *TaHSP70*, have function in stress tolerance and identifying stress related genes from this yeast screening is effective.

### Isolation and structural analysis of *TaPR-1-1*

**Figure 4:**
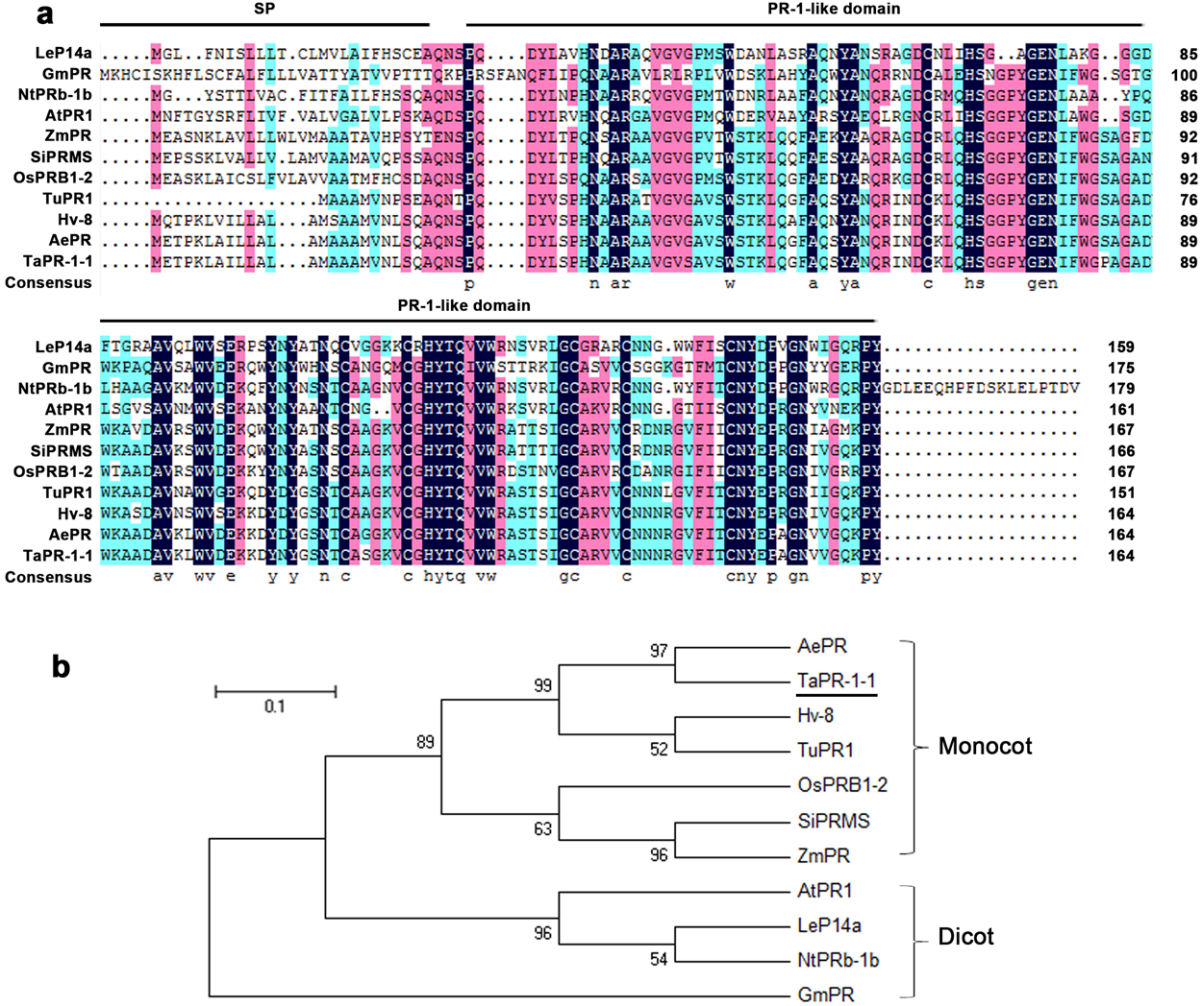
Protein structure and phylogenic tree of TaPR-1-1 and homologous PR proteins. (**a**) Amino acid sequence alignment of TaPR-1-1 protein and homologues in other plant species. Black shaded amino acids are the most conserved, red less, and blue least conserved. The positions of signal peptide (SP) and PR-1like domain are indicated by lines above the sequences, while consensus sequences are shown in lowercase letters below the sequences. (**b**) Phylogenic tree of TaPR-1-1 and homologous proteins. Abbreviations: At, *Arabidopsis thaliana*; Gm, *Glycine max*; Hv, *Hordeum vulgare*; Os, *Oryza sativa*; Zm, *Zea mays*; Le, *Lycopersicon esculentum*; Ae, *Aegilops tauschii*; Nt, *Nicotiana tabacum*; Tu, *Triticum urartu*; Si, *Setaria italica*; Ta, *Triticum aestivum*.

*TaPR-1-1* was selected for further study. Previous reports demonstrated that PR proteins participated in abiotic stress response in *Arabidopsis* and rice, but their functions in wheat were not clear. Four clones of *TaPR-1-1* were identified in all three treatments. Three clones contained an intron-less 495 bp coding region encoding a polypeptide of 164 amino acids with a predicted molecular mass of 17.6 kDa and pI of 8.57, whereas the fourth contained the same CDS, but lacked 72 bp at 5′ end corresponding to an N terminal 24 amino acids. Through ‘SignalP 4.1 Server’ analysis, this N terminal 24 amino acids was predicted to be a signal peptide (Supplementary Fig. S11). Protein analysis indicated that TaPR-1-1 protein, a member of TaPR-1 family group I, contained a signal peptide (SP) followed by a conserved PR-1-like domain (Fig. 4). TaPR-1-1 was aligned to the LeP14a protein used in resolving the first crystal structure of PR-1 family proteins. Most conserved residues were located in positions important for secondary structural folding, including four α-helices, a short 3_10_-helix, and a mixed, four-stranded β-sheet (Fig. 4a). Hence the three dimensional structure of TaPR-1 was similar to LeP14a. Phylogenetic tree analysis revealed that PR-1 protein family members could be divided into monocot and dicot groups, and TaPR-1-1 fell into the monocot group (Fig. 4b).

### Expression pattern of *TaPR-1-1* in wheat seedlings

**Figure 5:**
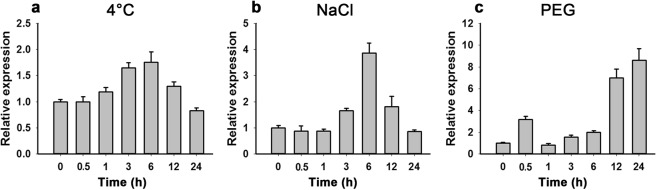
Expression patterns of *TaPR-1-1* in wheat seedlings subjected to different stress treatments. (**a**) Low temperature; two-week-old wheat seedlings were treated at 4 °C and samples were harvested at 0, 0.5, 1, 3, 6, 12 and 24 h. (**b**) Salinity; two-week-old wheat seedlings were treated at 250 mM NaCl and samples were harvested at 0, 0.5, 1, 3, 6, 12 and 24 h. (**c**) Osmotic stress; two-week-old wheat seedlings were treated with 16.1% PEG6000 and samples were harvested at 0, 0.5, 1, 3, 6, 12 and 24 h. *GAPDH* was used as the control. Three independent biological experiments, each with three technical replicates were performed. Error bars refer to 2× SE.

Many studies showed that *PR1* genes respond to pathogen attack. Real-time PCR was performed to identify expression patterns of the *TaPR-1-1* gene in 2-week-old wheat seedlings under various stress conditions. As shown in Fig. 5, *TaPR-1-1* expression was only slightly induced by low temperature (4 °C), but was strongly induced by NaCl and PEG. *TaPR-1-1* expression reached its highest level of 4-fold that of the control after exposure to salinity stress for 6 h. Expression of *TaPR-1-1* was also induced by osmotic treatment using PEG, and a double peak expression pattern was identified. The first small peak of about 4-fold the level of the control appeared after 0.5 h, then expression gradually increased until 12 h and 24 h when it reached 7-8-fold the control level. These results indicated that *TaPR-1-1* responded to abiotic stress.

### Overexpression of *TaPR-1-1* in *Arabidopsis* enhances freezing tolerance

**Figure 6:**
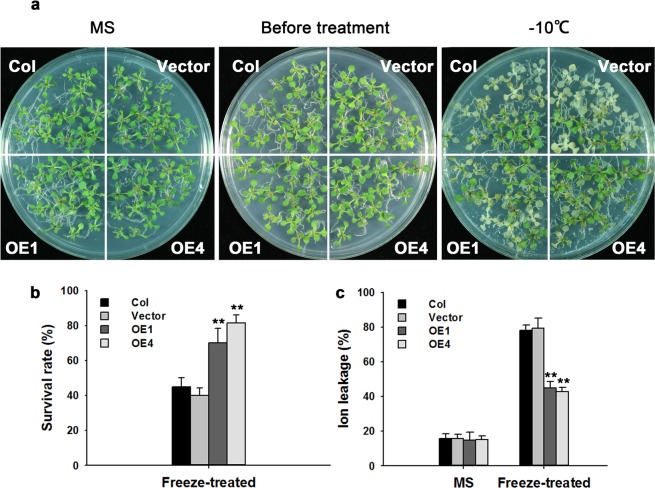
Overexpression of *TaPR-1-1* in *Arabidopsis* strengthens freezing tolerance. (**a**) Freezing tolerance phenotypes of *TaPR-1-1-OE* plants. The left panel shows two-week-old seedlings grown under normal condition, the middle panel shows two-week-old seedlings before treatment, and the right panel shows two-week-old seedlings subjected to −10 °C for 12 h. Photographs were taken following 3 days of recovery. (**b**) Survival rates for (**a**). (**c**) Ion leakage assays of the seedlings in (**a**). Three independent experiments were performed. Error bars refer to 2× SE. **P < 0.01 (Student’s *t*-test).

*TaPR-1-1* gene was cloned into expression vector pCAMBIA1300 with the 35S promoter, and more than 20 transgenic *Arabidopsis* lines were obtained. Expression levels of *TaPR-1-1* in 8 transgenic lines are shown in Supplementary Fig. S12; two transgenic lines, OE1 and OE4, with different expression levels of *TaPR-1-1* were selected for presentation of results. A previous study showed that PR1 proteins participated in freezing tolerance in winter rye^47^. Treated at −10 °C for 12 h, about 40% of the control seedlings were dead, whereas survival rates in the transgenic lines were more than 70% (Fig. 6a,b).

Cell membrane stability plays an important role in stress tolerance. Ion leakage used as a parameter of cell membrane stability was similar in control and transgenic seedlings under normal conditions, but following exposure to freezing stress the transgenic *TaPR-1-1* seedlings showed about 35% less ion leakage than those transformed with the empty vector and the Columbia wild type (Col) control, (Fig. 6c). Thus *TaPR-1-1-OE Arabidopsis* had more stable cell membranes than the control under freezing conditions, indicating that *TaPR-1-1* confers freezing tolerance.

### Overexpression of *TaPR-1-1* in *Arabidopsis* strengthens salinity tolerance

**Figure 7:**
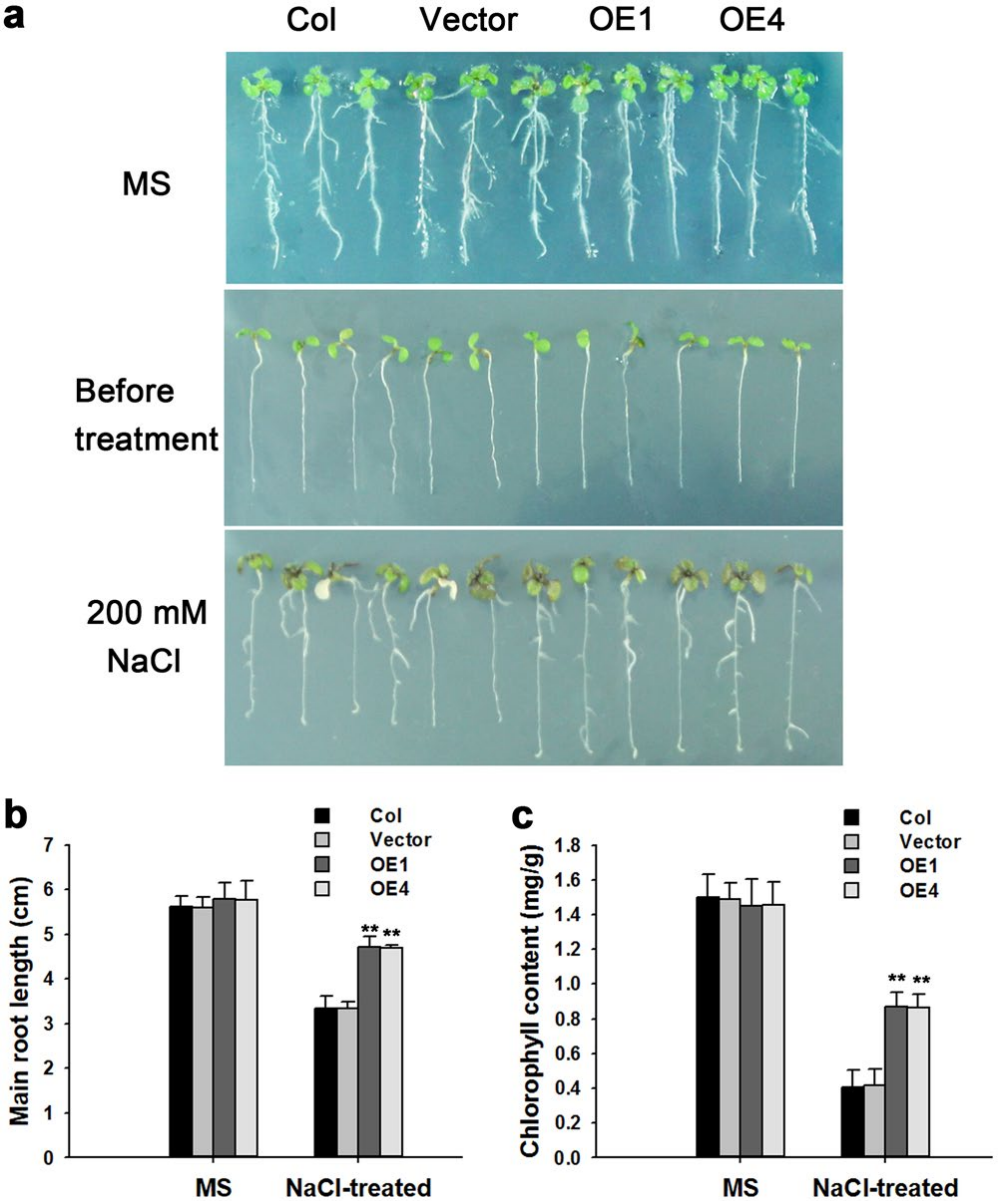
Overexpression of *TaPR-1-1* in *Arabidopsis* confers salinity tolerance. (**a**) Salinity tolerance phenotypes of *TaPR-1-1-OE* plants. 7-day-old *Arabidopsis* seedlings were transferred to MS solid medium and MS containing 200 mM NaCl. Photographs were made after 7 days. (**b**) Main root lengths for (**a**). (**c**) Chlorophyll contents for (**a**). Three independent experiments were performed. Error bars refer to 2× SE. **P < 0.01 (Student’s *t*-test).

*TaPR-1-1* was up-regulated by salinity treatment in wheat seedlings (Fig. 5b). Therefore possible salinity-tolerance in *TaPR-1-1-OE Arabidopsis* was further investigated. At the 7th day of growth on normal MS solid medium, transgenic and control *Arabidopsis* seedlings were transferred to MS solid medium containing 200 mM NaCl for another 7 days. The roots of transgenic plants under NaCl treatment were significantly longer than that of control (Fig. 7a,b).

Chlorophyll content is an important indicator of salt tolerance; with exposure to high salinity degradation of chlorophyll affects plant growth and yield^48^. There was no significant difference in chlorophyll content between transgenic and control plants under normal conditions, but in transgenic lines chlorophyll contents were significantly higher than in the control when exposed to high salinity for 7 days (Fig. 7c). Thus *TaPR-1-1-OE Arabidopsis* had stronger salt tolerance than control plants.

### Overexpression of *TaPR-1-1* in *Arabidopsis* confers osmotic tolerance

**Figure 8:**
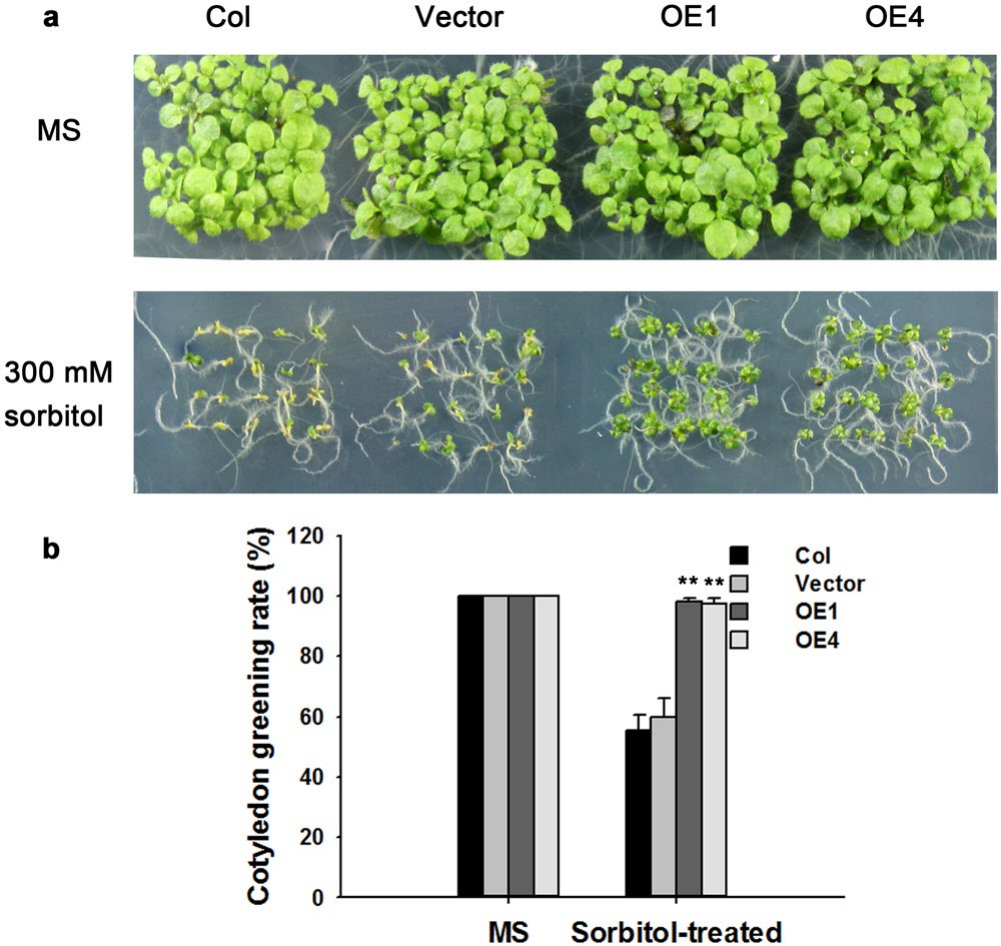
Overexpression of *TaPR-1-1* in *Arabidopsis* enhances osmotic tolerance. (**a**) Phenotypes of *TaPR-1-1-OE* and control plants. Seeds were planted on MS medium and MS medium supplemented with 300 mM sorbitol to induce osmotic stress. Photographs were taken after 7 days. (**b**) Cotyledon greening rates for (**a**). Three independent experiments were performed. Error bars refer to 2× SE. **P < 0.01 (Student’s *t*-test).

To study *TaPR-1-1* function under osmotic stress, *TaPR-1-1-OE* and control *Arabidopsis* were planted on MS media and MS media supplemented with 300 mM sorbitol simulating osmotic stress. As shown in Fig. 8, After 7 days of growth on sorbitol-supplemented MS medium almost 100% of transgenic plants had green cotyledons whereas comparative levels on the control were about 50%. This showed that *TaPR-1-1-OE Arabidopsis* had better osmotic tolerance than the control.

## Discussion

### Screening a wheat cDNA yeast library is an effective way to isolate functional genes

Although there are numerous methods to identify stress-related functional genes, screening a yeast library expressing heterologous cDNA is a further valid approach^49,50^. In this research, we isolated 4,695 genes responding to freezing, 2,641 genes responding to salinity, and 2,771 genes responding to osmotic stress from a cDNA yeast library. The effectiveness of screening was validated by characterizing the function when overexpressed *TaPR-1-1*, *TaMYC2* and *TaHSP70* in yeast and *TaPR-1-1* in *Arabidopsis*. Compared to map-based cloning and other methods of analysis, cDNA library screening is high throughput and capable of identifying many abiotic stress-related functional genes.

### Ubiquitin mediated protein degradation and stress tolerance

Through KEGG enrichment analysis, ubiquitin mediated proteolysis and proteasome pathways were enriched in overlapping genes (Fig. 2b). This implied that plants adjust to abiotic stress through degradation of cellular components. This requires the coordinated activities of enzymes, E1, E2 and E3. Some U-box E3 ubiquitin ligases have been shown to have roles in plant abiotic stress tolerance, such as AtPUB22 and AtPUB23 that participate in drought stress tolerance, AtPUB30 involved in salinity tolerance^51,52^, and AtSDIR1, AtPUB18 and AtPUB19 that modulate multiple stress responses^53,54^. However determination of which of E1, E2 and E3 takes part in stress tolerance and how they coordinate with each other needs further study.

### PR-1 function in wheat

Most studies on* PR-1* genes have focused on their function in biotic stress response^55^. Overexpression in *Arabidopsis* and yeast implied a role of *TaPR-1-1* in tolerance to freezing, salinity and osmotic stress (Figs 3, 6–8). Hence at least some PR-1 proteins have function in biotic and/or abiotic stress tolerance. Although expression profiles of some *TaPR-1* members were tested under pathogen attack, expression profiles of *TaPR-1* members under abiotic stress are still not adequately studied. Some evidence indicates that PR proteins, such as PR-2 (glucanase), PR-3 (chitinase) and PR-5 (thaumatin-like protein), inhibit microbial growth through enzymatic activity^56,57^. PR-1 proteins that contain a CAP-derived (cysteine-rich secretory protein, antigen 5, and pathogenesis-related-1) peptide 1 have been shown to confer stress tolerance^58^. However the actual mechanism of *TaPR-1-1* in abiotic stress tolerance and whether *TaPR-1-1* takes part in tolerance to both abiotic and biotic stress through the same pathway remains to be addressed.

## Conclusions

In this study, we screened a wheat cDNA yeast library, and identified 4,695 freezing-related genes, 2,641 salinity stress-related genes, and 2,771 osmotic stress-related genes. The gene function in stress tolerance was confirmed by study of an overlapping gene *TaPR-1-1* detected in all three stress treatments through overexpression in *Arabidopsis* and yeast. Together, this study provides insights on screening for stress-responsive functional genes that might be useful in dissecting and improving abiotic stress tolerance in crop plants.

## Supplementary information

Dataset
