# Supplementary material for: Identification of wheat stress-responding genes and TaPR-1-1 function by screening a cDNA yeast library prepared following abiotic stress
Source: Sci Rep. 2019 Jan 15;9:141. doi: 10.1038/s41598-018-37859-y (PMC6333785; doi:10.1038/s41598-018-37859-y)
Supplement: Supplementary file 1 — Dataset [file 41598_2018_37859_MOESM1_ESM.docx]

Identification of wheat stress-responding genes and *TaPR-1-1* function by screening a cDNA yeast library prepared following abiotic stress

Jingyi Wang, Xinguo Mao, Ruitong Wang, Ang Li, Guangyao Zhao, Jinfeng Zhao, Ruilian Jing^*^

National Key Facility for Crop Gene Resources and Genetic Improvement/Institute of Crop Science, Chinese Academy of Agricultural Sciences, Beijing 100081, China

* Correspondence: jingruilian@caas.cn

Contact Information

Ruilian Jing

Institute of Crop Science

Chinese Academy of Agricultural Sciences

Beijing 100081

P. R. China

Tel/Fax: 86-10-82105829


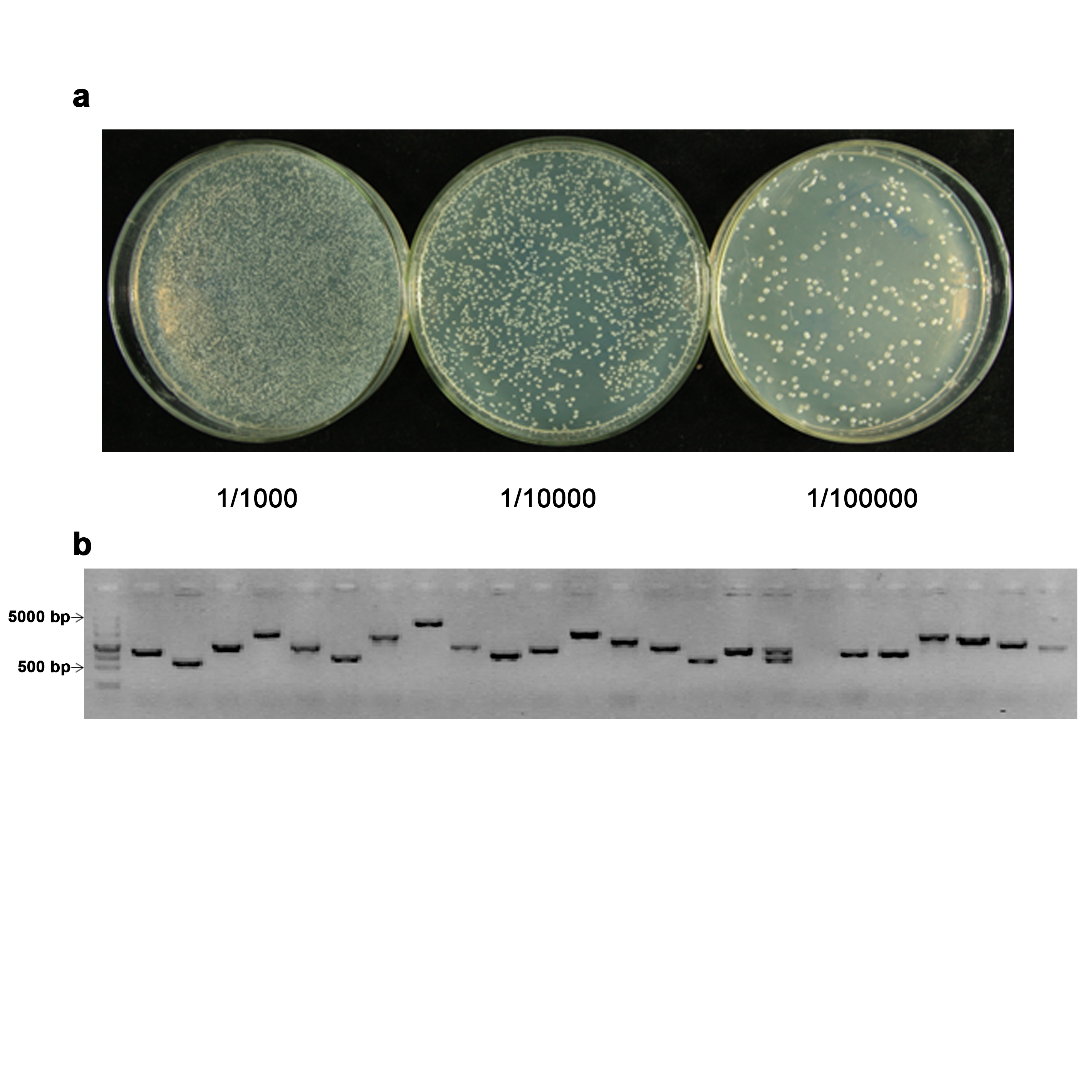


**Supplementary Figure S**1. Wheat cDNA library quality.

(a) 1/1,000, 1/10,000, 1/100,000 dilution yeast libraries incubated on SD/-Leu media. (b) Electrophoresis of 24 randomly selected yeast colony PCR products using T7 and 3' AD as primers.


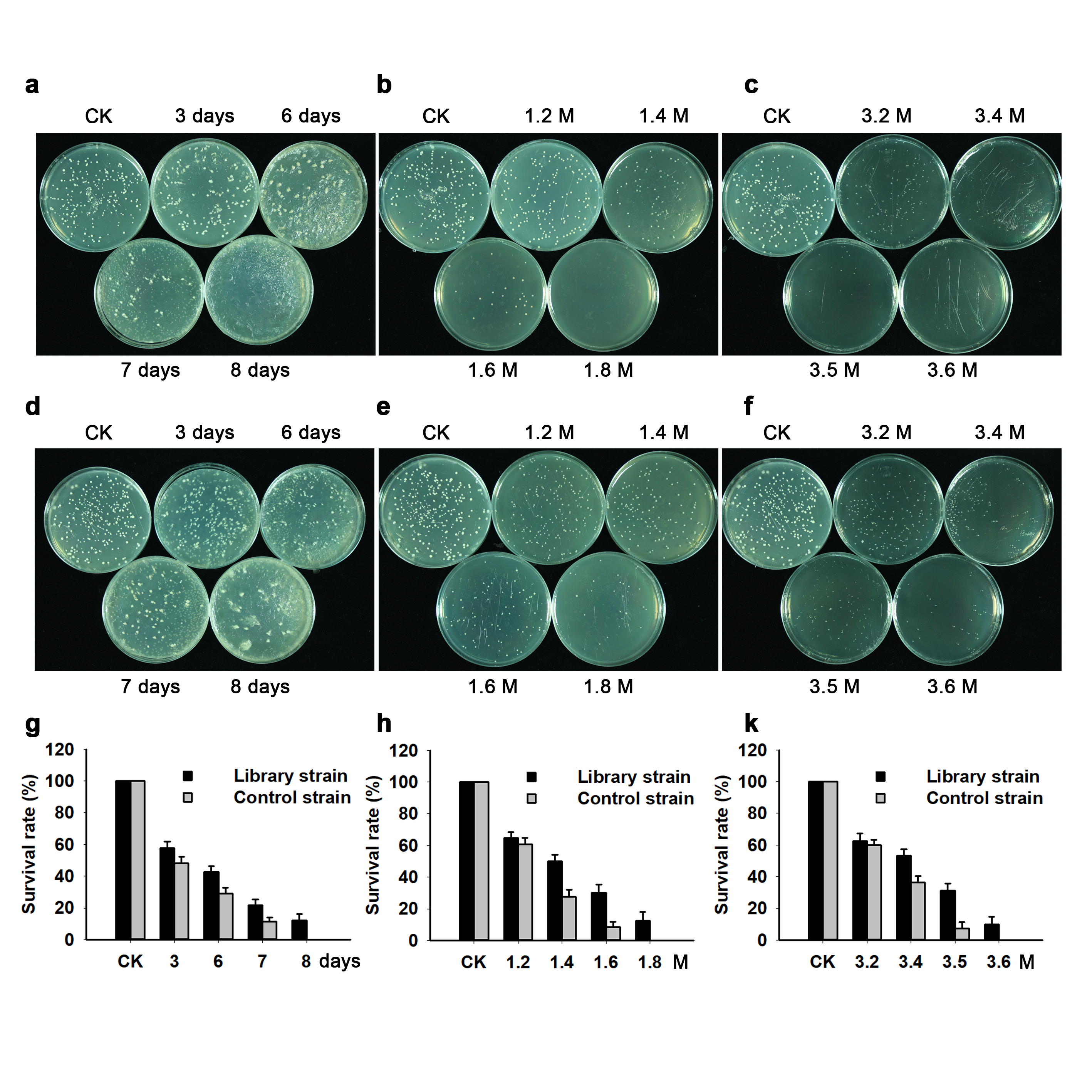


**Supplementary Figure S2.** Determination of screening conditions.

(a, d) 1/100,000 dilution yeast library strain (a) or control strain transformed with empty AD vector (d) grown on SD/-Leu medium for 3 days after -20°C treatment for 3 days, 6 days, 7 days, or 8 days. (b, e) 1/100,000 dilution yeast library strain (b) or control strain transformed with empty AD vector (e) grown on SD/-Leu medium supplemented with 1.2, 1.4, 1.6 or 1.8 M NaCl for 5 days. (c, f) 1/100,000 dilution library strain (c) or control strain transformed with empty AD vector (f) grown on SD/-Leu medium supplemented with 3.2, 3.4, 3.5, 3.6 M sorbitol for 5 days. (g-k) Survival rates for (a-f). Three independent experiments were performed. Error bars refer to 2× SE.


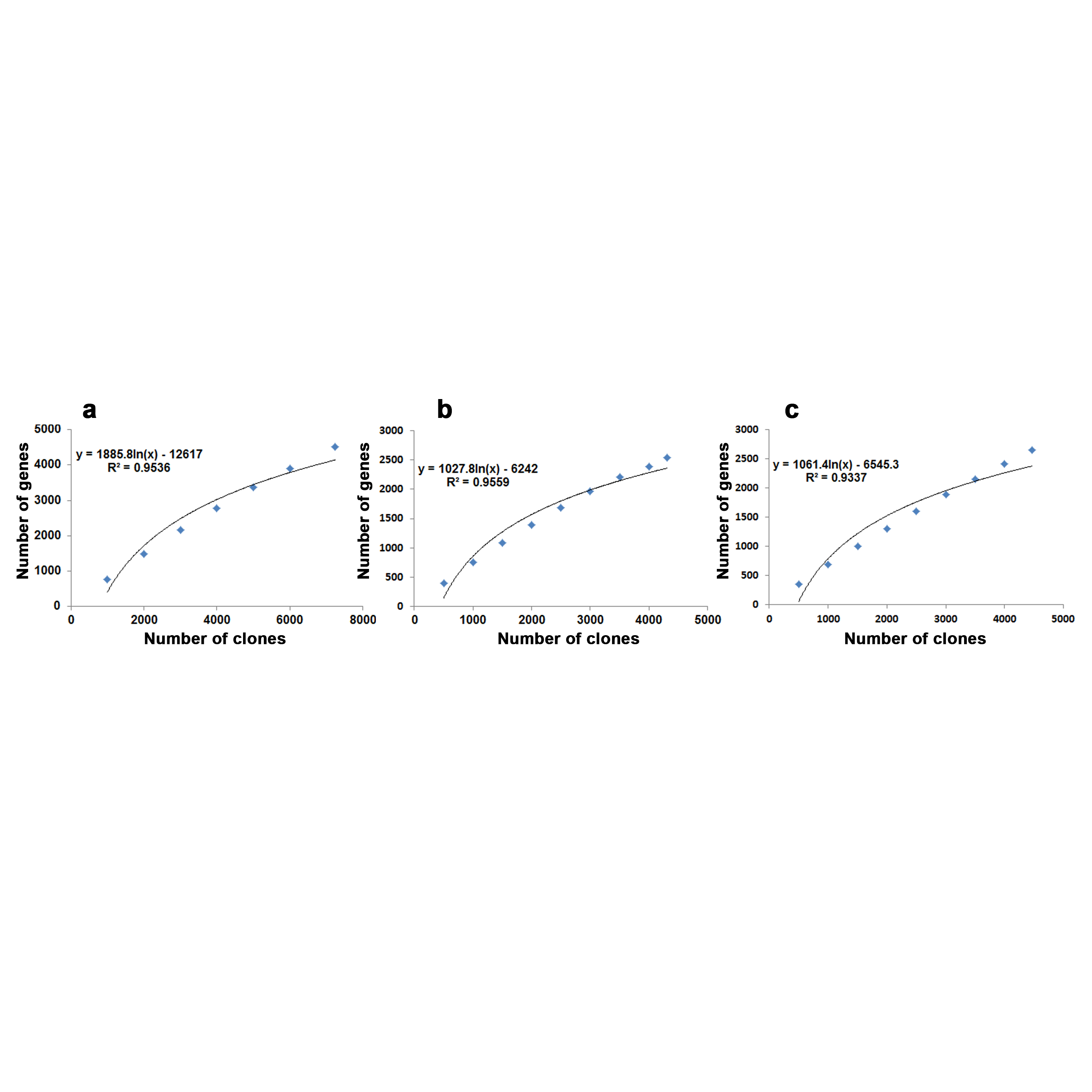


**Supplementary Figure S3.** Saturation curves for cDNA library screening.

Saturation curves for cDNA library screening under freezing (a), salinity (b) and osmotic (c) stress treatments. Data points indicate the true relationship between number of genes and number of clones, whereas the curves represent trend lines.


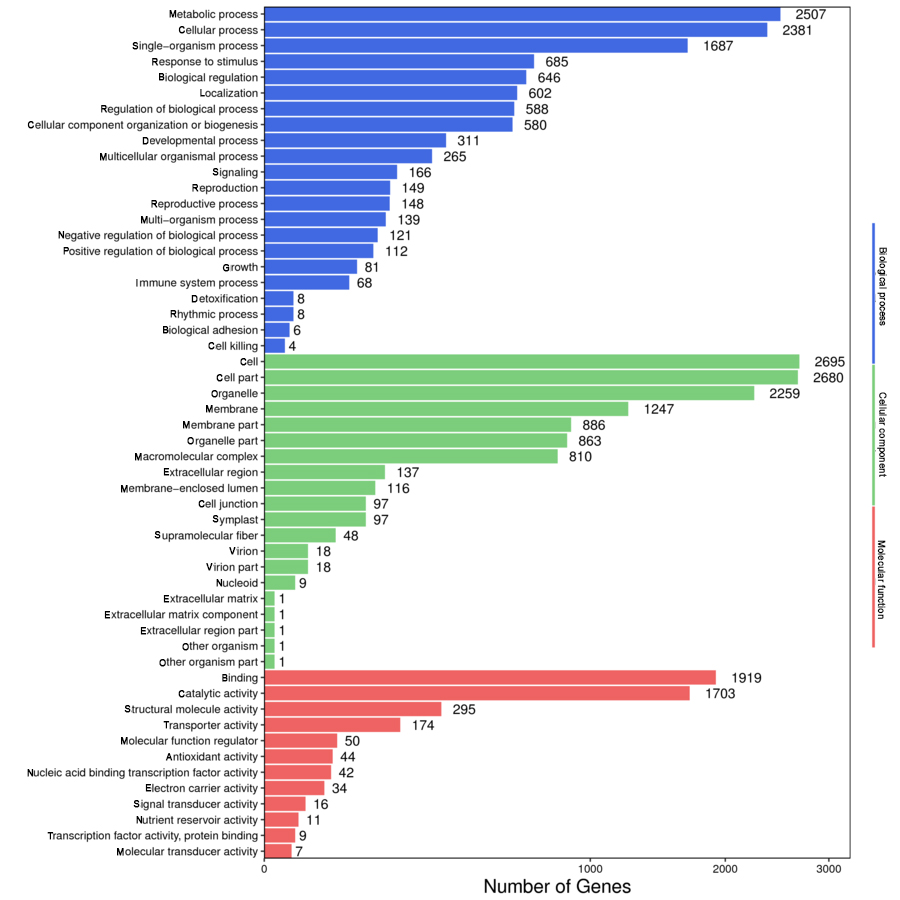


**Supplementary Figure S4.** Gene Ontology (GO) classification of genes isolated following freezing treatment.


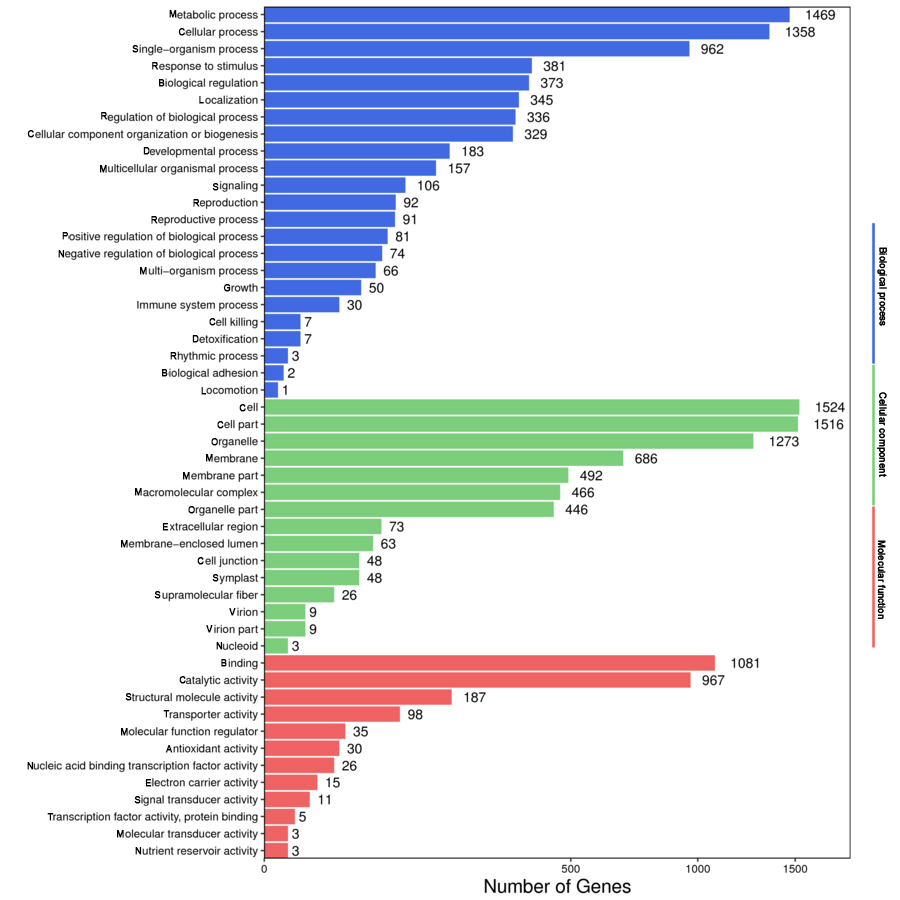


**Supplementary Figure S5.** Gene Ontology (GO) classification of genes isolated following salinity treatment.


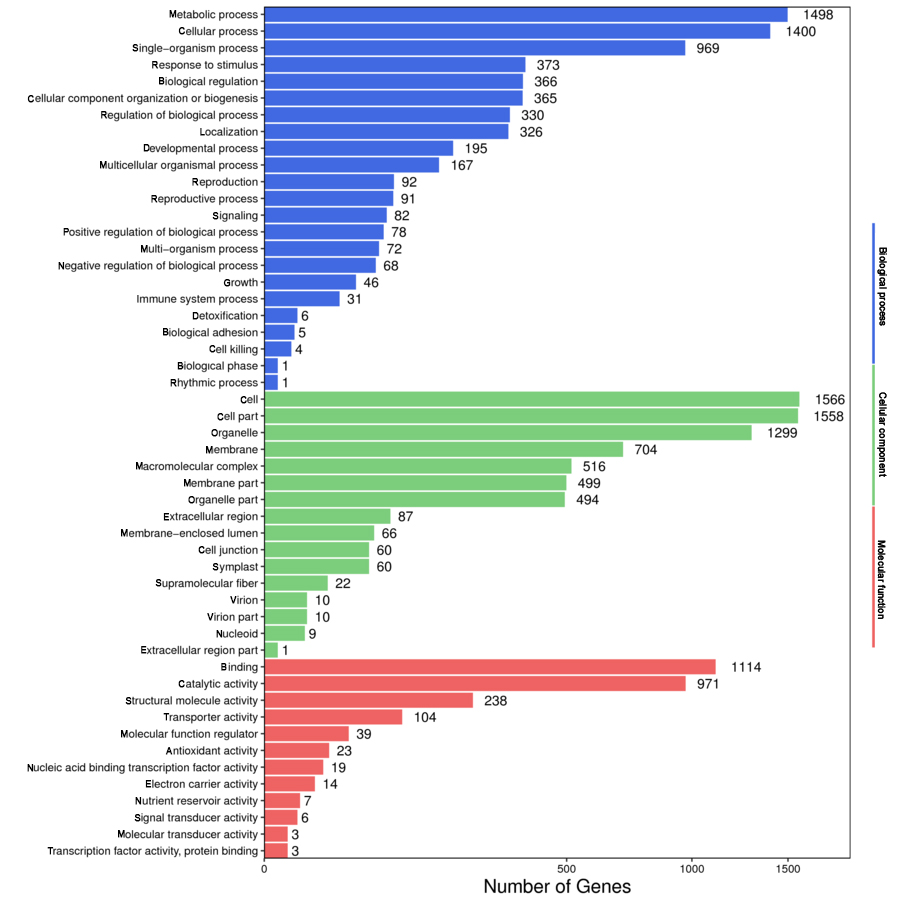


**Supplementary Figure S6.** Gene Ontology (GO) classification of genes isolated following osmotic stress treatment.


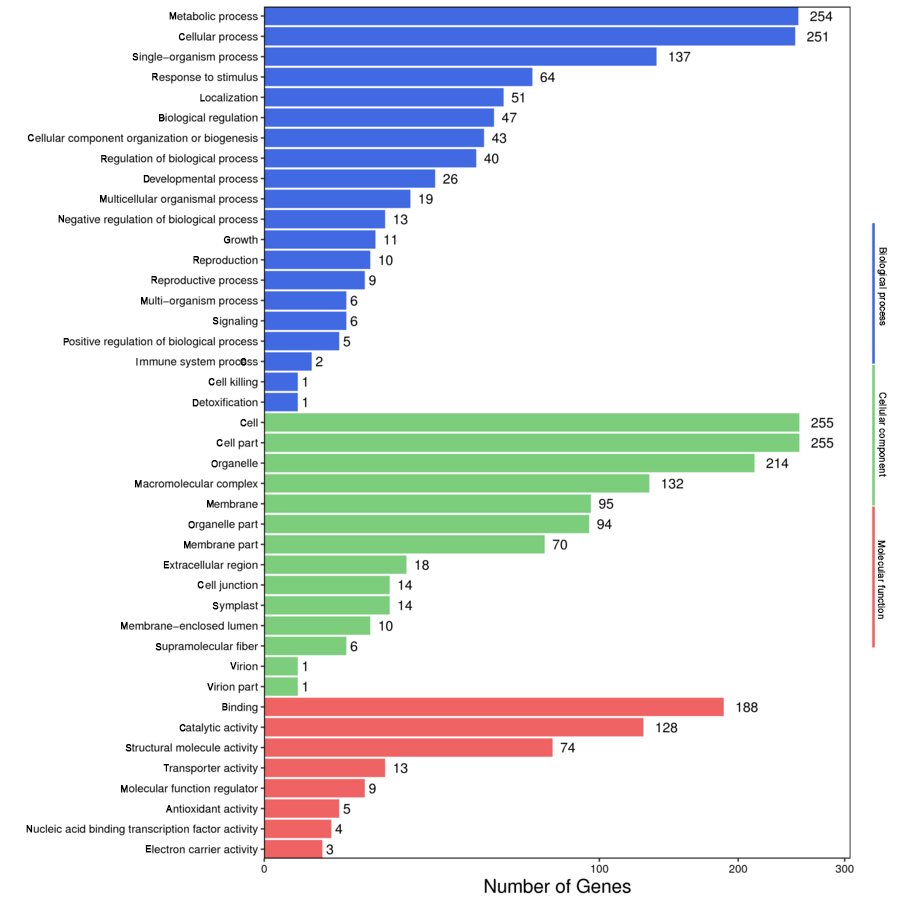


**Supplementary Figure S7.** Gene Ontology (GO) classification of genes isolated from the intersection region.


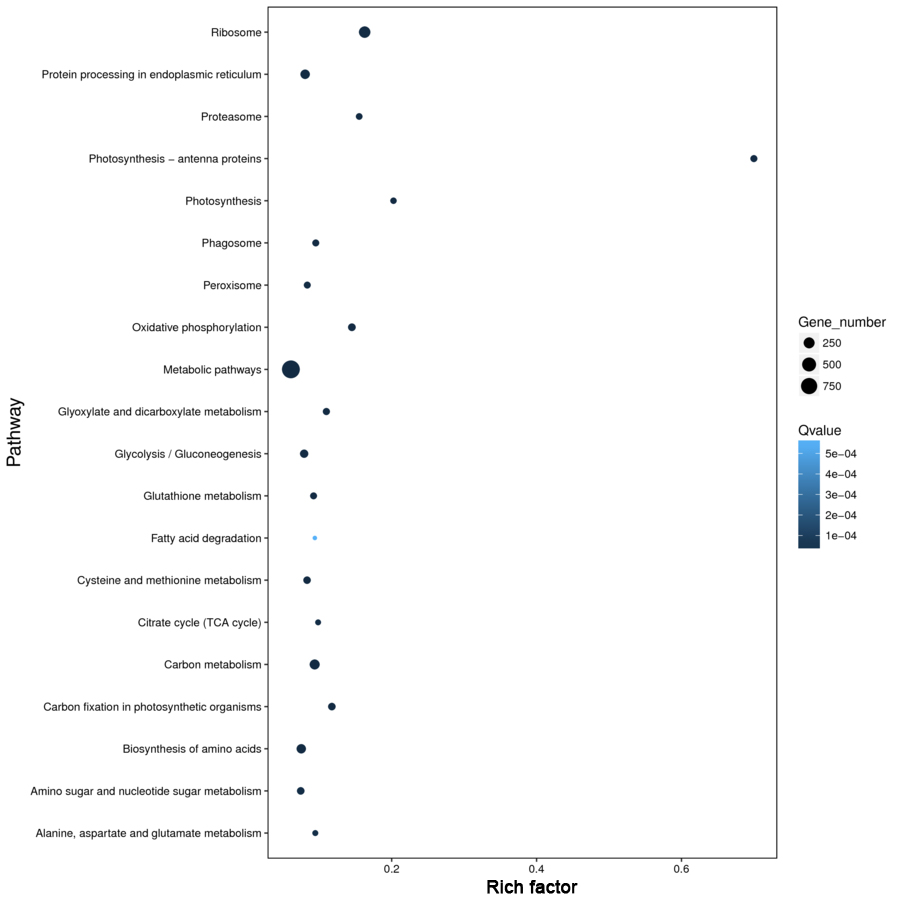


**Supplementary Figure S8.** KEGG pathway enrichment of genes isolated following freezing treatment. KEGG pathways for the isolated genes were retrieved (http://www.kegg.jp/kegg/kegg1.html). The rich factor reflects the proportion of isolated genes in a given pathway. Circle areas represent the relative numbers of isolated genes in the pathway; circle colors represent the range of Q values.


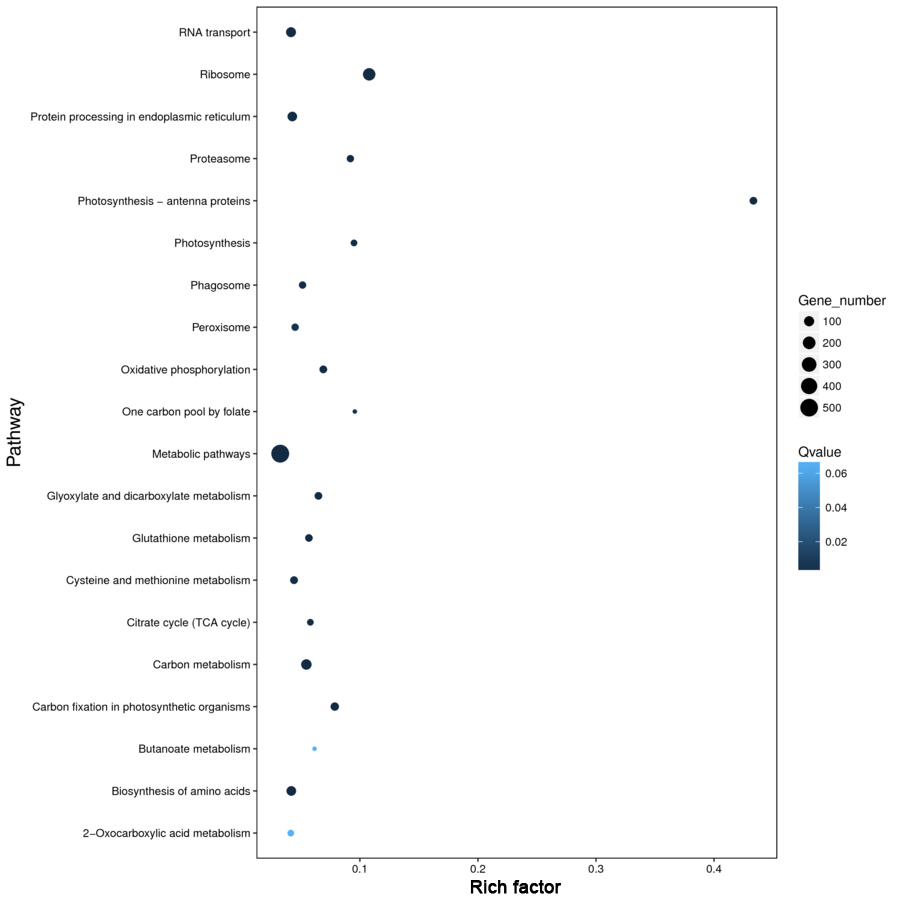


**Supplementary Figure S9.** KEGG pathway enrichment of genes isolated following salinity treatment. KEGG pathways for the isolated genes were retrieved (http://www.kegg.jp/kegg/kegg1.html). The rich factor reflects the proportion of isolated genes in a given pathway. Circle areas represent the relative numbers of isolated genes in the pathway; circle colors represent the range of Q values.


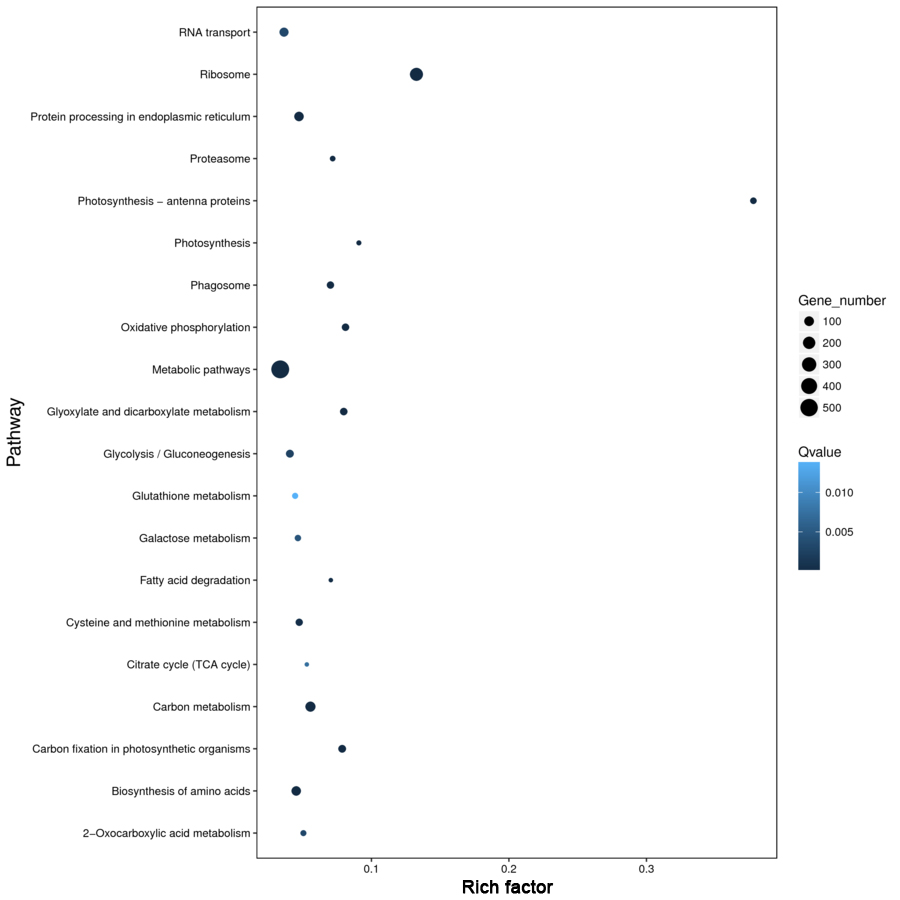


**Supplementary Figure S10.** KEGG pathway enrichment of genes isolated following osmotic stress treatment. KEGG pathways for the isolated genes were retrieved (http://www.kegg.jp/kegg/kegg1.html). The rich factor reflects the proportion of isolated genes in a given pathway. Circle areas represent the relative numbers of isolated genes in the pathway; circle colors represent the range of Q values.


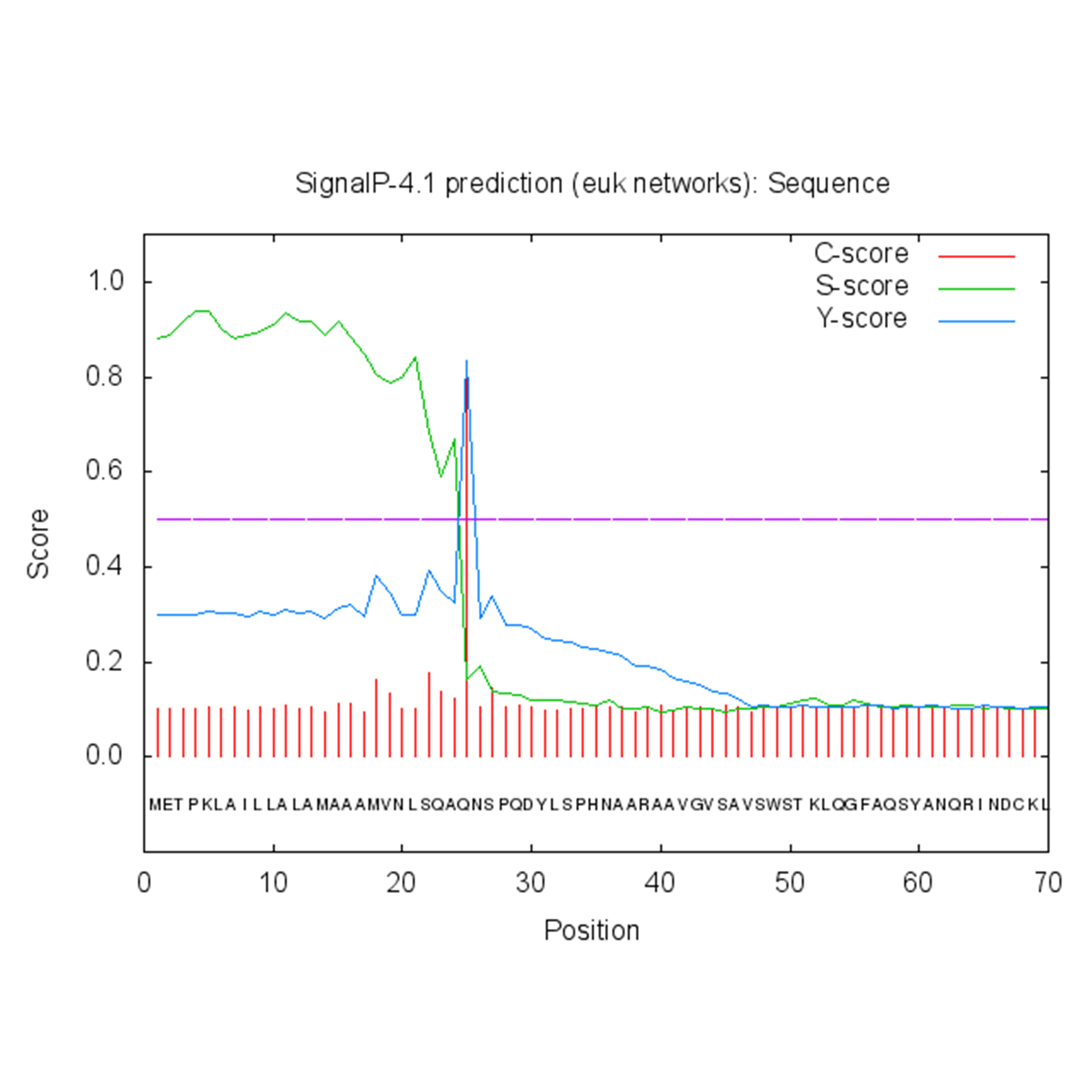


**Supplementary Figure S11.** TaPR-1-1 protein signal peptide prediction by “SignalP 4.1 Server” analysis.


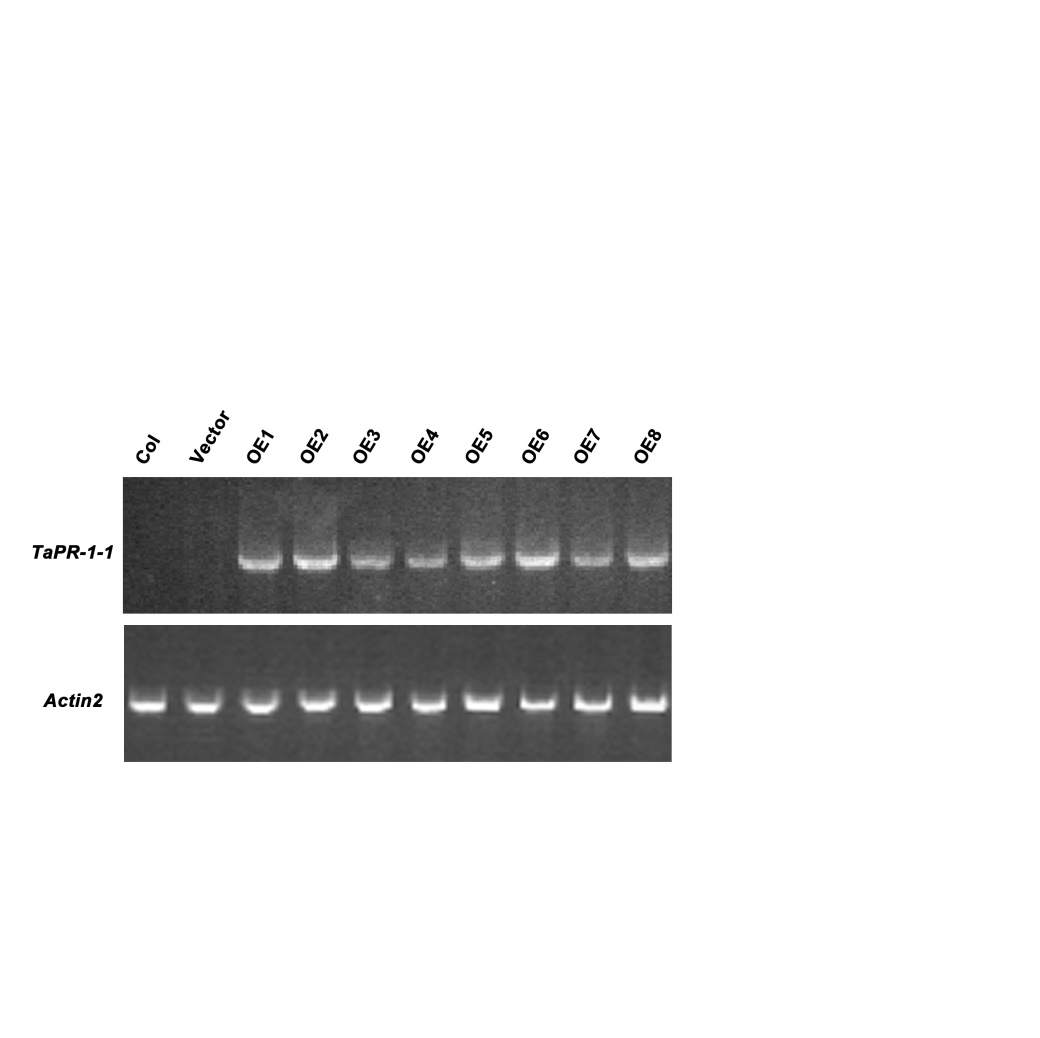


**Supplementary Figure S12.** Semi-quantitative PCR detection of *TaPR-1-1* expression levels in *TaPR-1-1-OE Arabidopsis* lines.

**Supplementary Table S1.** Adapter information used for vector construction

| **Reading frame** | **Adapter sequence (5' to 3')** |
| --- | --- |
| Frame α | TCGTCGGGGACAACTTTGTACAAAAAAGTTGG |
|  | CCAACTTTTTTGTACAAAGTTGTCCCC |
| Frame β | TCGTCGGGGACAACTTTGTACAAAAAAGTTGGA |
|  | TCCAACTTTTTTGTACAAAGTTGTCCCC |
| Frame γ | TCGTCGGGGACAACTTTGTACAAAAAAGTTGGAA |
|  | TTCCAACTTTTTTGTACAAAGTTGTCCCC |

**Supplementary Table S2.** Primers used for vector construction

| **Primer name** | **Primer sequence (5' to 3')** | **Vector** | **Restriction enzyme** |
| --- | --- | --- | --- |
| TaPR-1-1-F1 | CTAGACTAGTACACCGAACCAGGAAGTAATGGAG | pCAMBIA1300 | *Spe* I |
| TaPR-1-1-R1 | TGACGGTACCGTATGGTTTCTGTCCAACAACATTCCC | pCAMBIA1300 | *Kpn* I |
| TaPR-1-1-F2 | CTAGCATATGACACCGAACCAGGAAGTAATGGAG | pGBKT7 | *Nde* I |
| TaPR-1-1-R2 | TGACGGATCCGTATGGTTTCTGTCCAACAACATTCCC | pGBKT7 | *Bam*H I |
| TaMYC2-F | CTAGCATATGGAGCAAAGCAGAGCAATGGAGTCC | pGBKT7 | *Nde* I |
| TaMYC2-R | TGACGGATCCCTCACATTGAGATGACCGAGTGAGACTG | pGBKT7 | *Bam*H I |
| TaHSP70-F | CTAGCATATGCTCGATCTAGCTAGAGCCATGGCG | pGBKT7 | *Nde* I |
| TaHSP70-R | TGACGGATCCCTTAGTCGACCTCCTCGATCTTGGG | pGBKT7 | *Bam*H I |

**Supplementary Table S3.** Primers used for real-time PCR and semi-quantitative PCR

| **Primer name** | **Primer sequence (5’ to 3’)** |
| --- | --- |
| TaPR-1-1-RT-F | GAACCAGGAAGTAATGGAGACGCCC |
| TaPR-1-1-RT-R | CAGGTGTTGGACCCGTAGTTGTAGTC |
| GAPDH-RT-F | CTGCATCATACGATGACATC |
| GAPDH-RT-R | TGTCACCGACAAAGTCAGTG |
| Actin2-F | AGCACTTGCACCAAGCAGCATG |
| Actin2-R | ACGATTCCTGGACCTGCCTCATC |
